# Prevention of occlusal caries using Vanish^TM^ XT: an 18-month follow-up randomized clinical trial

**DOI:** 10.1186/s12903-024-05095-8

**Published:** 2024-11-01

**Authors:** Alaa Baik, Najlaa Alamoudi, Osama Felemban, Azza El-Housseiny, Eman Almabadi, Khadijah Baik, Amani Altuwirqi, Ibrahim Masoud

**Affiliations:** 1 Pediatric Dentistry Department, King Abdulaziz University Dental Hospital, Jeddah, 21589 Saudi Arabia; 2 Pediatric Dentistry Department, Faculty of Dentistry, King Abdulaziz University, P.O. Box 80209, Jeddah, 21589 Saudi Arabia; 3 Pediatric Dentistry Department, Faculty of Dentistry, Alexandria University, Alexandria, 21526 Egypt; 4 Department of Preventive Dental Sciences, Taibah University Dental College & Hospital, Al-Madinah Al-Munawwrah, 42353 Saudi Arabia; 5 Oral and Maxillofacial Prosthodontics Department, Faculty of Dentistry, King Abdulaziz University, Jeddah, 21589 Saudi Arabia; 6 JedMed Health Center, Jeddah, Saudi Arabia

**Keywords:** Randomized clinical trial, Vanish^TM^ XT Varnish, Occlusal caries, Fissures sealant, Newly erupted first permanent molars

## Abstract

**Aim:**

To evaluate the effectiveness of a light curable resin-modified glass ionomer varnish (Vanish^TM^ XT) in the prevention of occlusal caries compared to topical fluoride varnish in newly erupted first permanent molars over 18 months.

**Methods:**

A randomized controlled clinical trial was conducted using a split-mouth design. A total of 53 participants aged 6–9 years with 97 pairs of caries-free newly erupted first permanent molars were enrolled in the study. Each molar in the pair was randomly assigned either to the experimental group, which received the Vanish^TM^ XT Extended Contact Varnish, or to the control group, which received topical fluoride varnish (Vanish^TM^ 5% Sodium Fluoride White Varnish). Follow-ups were performed at 6, 12, and 18 months to evaluate dental caries development.

**Results:**

At the 6-month follow-up, caries development was significantly higher in the control group (7.8%) than that in the experimental group (0%) (*P* = 0.031). At the 12-month follow-up, significantly (*P* = 0.012) more occlusal caries developed in the control group (12.2%) compared to the experimental group (1.2%). At the 18-month follow-up, significantly (*P* = 0.002) more occlusal caries developed in the control group (14.3%) compared to the experimental group (1.1%).

**Conclusion:**

Compared to fluoride varnish, Vanish^TM^ XT was significantly more effective in preventing caries on the occlusal surfaces of newly erupted first permanent molars at 6, 12, and 18 months.

**Trial registration:**

Registration number at ClinicalTrials.gov: NCT04579536 on 08/10/ 2020, retrospectively registered.

## Background

Despite ongoing efforts to prevent and treat dental caries, it remains a chronic disease prevalent in both children and adults. In permanent dentition, occlusal surfaces of molars with deep pits and fissures are most likely to develop caries, whereas smooth surfaces in the labial and lingual regions are the least likely to develop caries. In children aged 5–17 years, 67–90% of caries are located on the occlusal surfaces of permanent molars [1]. Newly erupted first permanent molars are highly prone to occlusal caries [2, 3] as these can be difficult to clean, retaining debris in their deep fissures. Recently, the association between the occlusal caries development in permanent second molars and the occlusal surface level at different eruption stages has been investigated. A significant increase in caries development in molars during their eruption stages, before reaching the occlusion level, has been found compared to molars that reached full occlusion [4].

Fissure sealants are used as physical barriers to seal deep pits and fissures. These are effective in preventing occlusal caries. However, their efficacy in caries prevention depends on the sealant retention. Resin-based sealants require effective operative field isolation and etching before application; thus, their success highly depends on the application technique and patient cooperation. The etching and drying steps are critical and can adversely affect the longevity of sealants if not performed properly [5].

Moisture-friendly materials, such as glass ionomer cement (GIC), have been used to prevent caries development in newly erupted molars when effective operative field isolation is challenging. However, systematic reviews and meta-analyses regarding the effectiveness of GIC in preventing occlusal caries development remain inconclusive [5]. Another studied alternative has been fluoride varnish. However, it remains on the tooth surface for a short time or until the patient brushes their teeth [5].

Recently, the Vanish^TM^ XT Extended Contact Varnish (3 M ESPE, St. Paul, MN, U.S.A) was introduced into the market. Vanish^TM^ XT is a light-cured resin-modified glass ionomer (RMGI) varnish [6]. Vanish^TM^ XT is less sensitive to moisture and can slowly release fluoride, calcium, and phosphate ions into the oral cavity. It can also be used as a sealing material on tooth surfaces exposed to acid erosion and around orthodontic devices to re-mineralize white spots [7]. Based on the manufacturer assumption, Vanish^TM^ XT can be used as a protective layer on the occlusal surface of newly erupted molars for up to 6 months.

Studies investigated the effects of these light-curable RMGI varnishes on the prevention of occlusal caries in newly erupted permanent first molars were limited. Therefore, this split-mouth study aimed to evaluate the effectiveness of Vanish^TM^ XT in the prevention of occlusal caries for up to 18 months in comparison to a fluoride varnish in newly erupted first permanent molars. We hypothesized that, within each pair, the probability of developing dental caries on the fluoride varnis tooth, while the Vanish^TM^ XT tooth remains sound, is greater than the probability of dental caries developing on the Vanish^TM^ XT tooth, while the fluoride varnish tooth remains sound.

## Methods

This was a split-mouth, randomized, controlled clinical trial over 18 months, with follow-ups at 6, 12, and 18 months. This study was approved by the Committee of Research Scientific Units of King Abdul-Aziz University, Faculty of Dentistry, Jeddah, Saudi Arabia (reference no. 77–0319). Additionally, it was retrospectively registered at ClinicalTrials.gov on 08/10/ 2020 under the registration number NCT04579536. The study followed the guidelines published by the Consolidated Standards of Reporting Trials [8].

### Participants

For Sample size calculation, assuming an 18-month success rate of 98.3% for ‟Vanish^TM^ XT” varnishand 88.9% for FV [9] and using the function of inequal proportion of two dependent groups (McNemar test) in G*Power software (version 3.1.9.2), it was found that a sample of 70 pairs of first permanent molars was required to detect a statistical difference of an effect size of 10% between the groups at the significance level of 0.05 with a power of 95%. To account for loss to follow up, 20% (14 pairs) were added to the sample size. The total sample was composed of 84 pairs of partially erupted first permanent molars.

Participants were screened at the pediatric dentistry clinics at King Abdul-Aziz University Dental Hospital, Jeddah, Saudi Arabia, from September 2019 to February 2020. Detailed information about the trial objectives and methodology was provided to the participants’ parents. A total of 105 participants aged 6–9 years who attended pediatric dental clinics were assessed for eligibility according to the following inclusion criteria: healthy children at high risk of dental caries (with no exposure to fluoride, having proximal caries in one or more teeth other than the intervention teeth, or having more than three times exposure to fermentable carbohydrates per day, having between meals snacks, or low socioeconomic status) [10], with a minimum of one pair of contralateral newly erupted first permanent molars with deep pits and fissures free from caries, restoration/sealants, cracks, and all four first permanent molars present in the oral cavity. Clinical examination assessed carious lesions at baseline according to ICDAS II criteria, and only teeth with an ICDAS II score of zero were included in this study. Bitewing radiographs were obtained for each child to detect proximal caries according to the protocol for radiographic examination of patients at high-risk. Newly erupted first permanent molars were included if the occlusal surface of the molar was partially or completely erupted and more than half of the facial surface was covered with gingival tissue [11].

Exclusion criteria comprised participants whose parents refused to sign the consent form, children with known acrylate allergies, and uncooperative children. Permanent first molars with proximal caries or full occlusion were also excluded. No children were excluded based on their sex, race, or socio-economic background. Fifty-six participants met the eligibility criteria and were invited to participate in the study, among which 53 agreed to participate in the study and were included. The procedures and possible benefits and risks were explained to the participants’ parents, and written informed consent was obtained.

### Examiner calibration

To achieve good inter- and intra-examiner reliability in caries detection using the International Caries Detection and Assessment System (ICDAS-II) and radiographic examination, training and calibration were performed.

To calibrate the examiners (two pediatric dentists and one general dentist), the first permanent molars of 20 children who were not included in the study were randomly chosen and examined clinically (according to ICDAS II criteria) and radiographically. Each evaluator independently scored each tooth. For intra-examiner reliability, the teeth were re-examined after 14 days, and the levels of consistency between the two readings were assessed. The results produced by the evaluators were analyzed using Cohen’s kappa. Intra-examiner reliability showed almost perfect agreement with 1 for the radiographic examination and 0.9 for the clinical assessment [12]. The kappa values for inter-examiner reliability were in perfect agreement with 1 for the radiographic examination and 0.88 for the clinical examination.

### Randomization and treatment allocation

Once the participants were included, randomization of the treatments was performed. It was designed as blocked randomization to ensure a balanced distribution of treatment materials on both sides. Each block contained two “As” and two “Bs” in different orders. The unit of randomization was the side of the mouth. The letter “A” means that Vanish^TM^ XT was used on the right side and FV was applied to the left side. The letter “B” means that Vanish^TM^ XT was used on the left side and FV was applied to the right side. A random sequence number was generated and used to create a sequence of group assignments corresponding to the block number. The assignment sequence was concealed from the operator and maintained by an assistant who was not involved in the study procedures until the intervention visit. Using a split-mouth design, pairs of maxillary or mandibular first molars were included so that one side was randomly allocated to the Vanish^TM^ XT group (Vanish^TM^ XT varnish, 3 M ESPE, Dental Products, St. Paul, MN, U.S.A.) and the contralateral side to Fluoride Varnish (FV) group (Vanish^TM^ 5% Sodium Fluoride White Varnish, 3 M ESPE, Dental Products, St. Paul, MN, USA).

### Interventions

At the baseline visit and after randomization, all clinical steps were performed by the principal operator. Teeth prophylaxis was performed using a rotating cup without paste. The teeth were isolated using cotton rolls and a saliva ejector. After isolation, the teeth were thoroughly dried. The materials were then applied according to the manufacturer’s instructions to the deep pits and fissures of the occlusal surface of the newly erupted first permanent molars.

In the Vanish^TM^ XT tooth, etching was performed for 15 s with 35% phosphoric acid (Scotchbond Universal Etchant; 3 M ESPE, St Paul, MN, USA), followed by thorough rinsing for 60 s and drying for 5 s using air syringe. One click of the dispenser containing the Vanish^TM^ XT (3 M ESPE, Dental Products, St. Paul, MN, U.S.A.) with an equal amount of liquid/paste was dispensed onto a mixing pad, followed by 15 s of mixing. The Vanish^TM^ XT was applied on the occlusal and buccal/lingual surfaces in a thin layer (0.5 mm or less) using a brush and subsequently light-cured for 20 s. The material was meticulously inspected after curing to ensure complete setting and retention before the child was dismissed.

In the FV tooth, the 5% Sodium Fluoride White Varnish with tricalcium phosphate (3 M ESPE, Dental Products, St. Paul, MN, USA) was used. After tooth cleaning, a thin layer of the FV was applied using an applicator brush to control the occlusal and tooth surfaces. All remaining teeth received the same FV application as a preventive standard of care measure. In order to mitigate any potential interaction effects between FV and Vanish XT, we made efforts to minimize the exposure of experimental teeth (Vanish XT) to FV. This was achieved by isolating these teeth using cotton rolls and saliva ejectors while applying FV to other tooth surfaces. Additionally, diet counseling and oral hygiene instructions using a dental model, toothbrushes, and dental floss were provided to the child and caregiver.

### Follow-up evaluation

The outcome of the study was the development of new caries (defined as ICDAS II score of 1,2, or 3) on the occlusal surfaces of the first molars after 6, 12, and 18 months since the varnish application. Two trained and calibrated examiners performed a new visual caries examination using the ICDAS-II criteria. A rotating toothbrush was used to clean the occlusal surface of debris and plaque. Thereafter, the occlusal surface was dried and clinical examination was performed. Bitewing radiographs were taken at 6 months intervals to monitor the occlusal and proximal surface conditions as a part of the follow-up radiographic examination recommendations for patients at a high risk of caries. Any carious lesions in the study molars received appropriate restorations and treatment and were recorded as a failure. At each follow-up visit at 6, 12, and 18 months, the teeth received the same baseline treatment according to their group allocation. Reapplication of the material was performed at each follow up visit after evaluating teeth surfaces. All the other teeth received FV as a preventive standard of care. The children and their parents were instructed to continue their usual oral hygiene regimens.

### Blinding

Blinding of the operator was difficult at the time of the materials application because of the nature of the treatment. However, the examiners were blinded to the type of material used on each side of the first permanent molars during the follow-up visits when they performed the clinical evaluation and when they evaluated the bitewing radiographs at 6, 12, and 18 months of follow-up.

### Statistical analysis

The demographic data and oral health status of the participants were analyzed through univariate analysis. To account for the split-mouth design of the study, the success in the two groups of Vanish^TM^ XT (experimental tooth) and FV (control tooth) were compared at 6-, 12-, and 18-month follow-up using the McNemar’s test. The data were processed using the SPSS software version 20. The significance level was set at *P* < 0.05.

## Results

In total, we included 53 (50.48%) eligible participants whose parents agreed on their participation in the study out of the total 105 screened children. The 53 participants had 97 pairs of caries-free, newly erupted permanent first molars that were included in the trial.

### Demographic characteristics of the study sample

The final sample comprised 53 children of both sexes, with an age range of 6–9 years (mean age 7.19 ± 0.86 years). Overall, more males (56.6%) were enrolled in the trial than females (43.4%). The most common education level was college/university for both fathers (66.0%) and mothers (58.5%) (Table 1). Approximately half of the pairs were in the maxillary arch (51.5%), and 48.5% were in the mandibular arch.

**Table 1:** Proportion distribution of demographic data and characteristics of the participants at baseline

Demographics	*N*	Mean ± SD
**Age**	53	7.19 ± 0.86
**dmft/DMFT**		7.11 ± 3.5
**Total**		**N (%)** **53 (100%)**
**Nationality**	Saudi	39 (73.6%)
	Non-Saudi	14 (26.4%)
**Sex**	Male	30 (56.6%)
	Female	23 (43.4%)
**Fathers’ highest level of education**	College/University	35 (66.0%)
	High school	13 (24.5%)
	Less than high school	5 (9.5%)
**Mothers’ highest level of education**	College/University	31 (58.5%)
	High school	18 (34.0%)
	Less than high school	4 (7.5%)
**Frequency of number of pairs included per participant**	One pair (two teeth)	9 (17.0%)
	Two pairs (four teeth)	44 (83.0%)

N: Total number of participants. SD: standard deviation

### Lost to follow-up

**Fig. 1:**
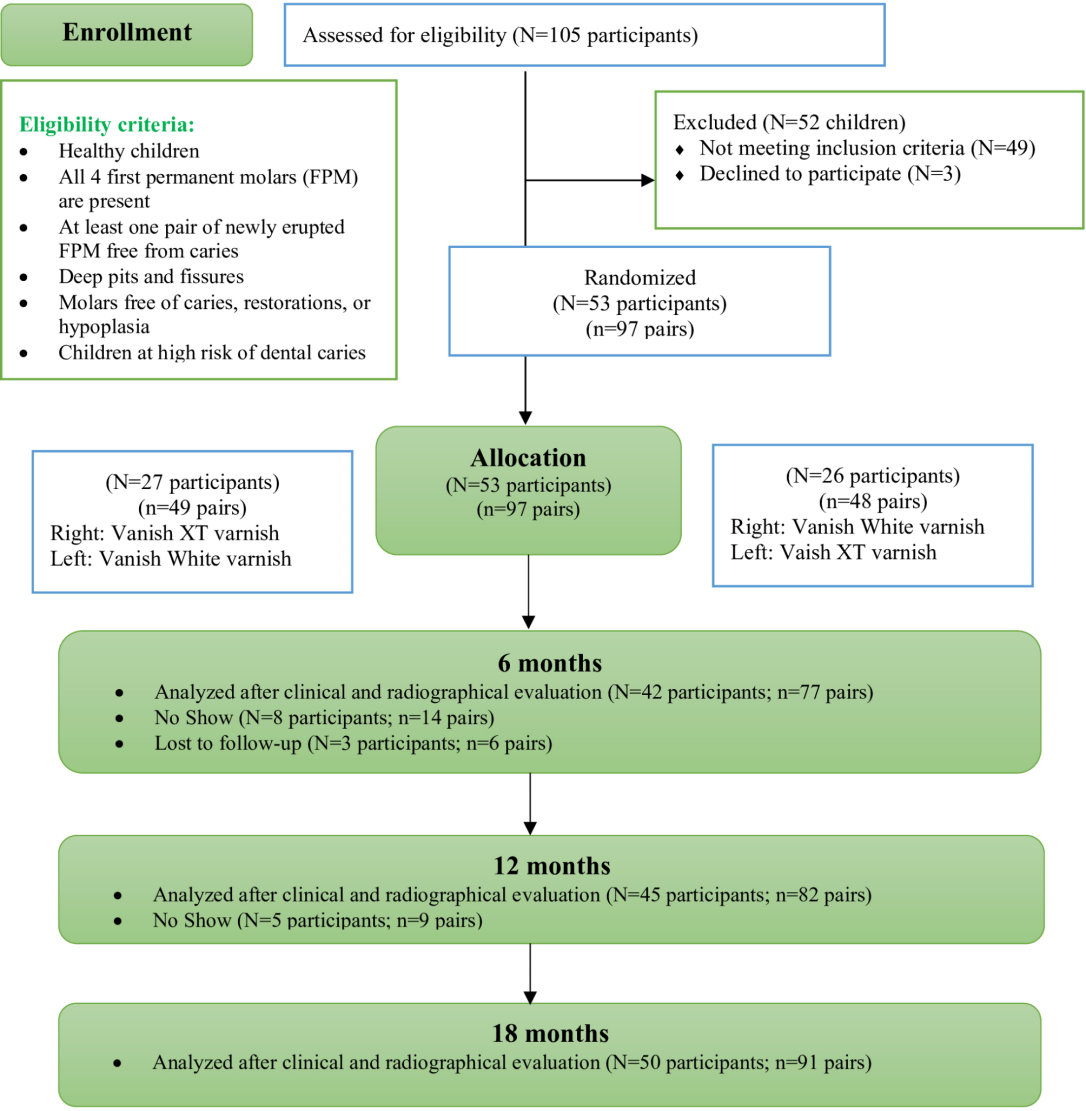
Study flow diagram up to 18 months of follow-up. N: number of participants; n: number of pairs; FPM: first permanent molar

At the 6-month follow-up, 42 participants (77 pairs) were assessed and analyzed. The parents of eight participants (14 pairs) did not attend because of the COVID-19 nationwide lockdown in Saudi Arabia at the time. Three participants (six pairs) ignored all attempts to contact them and were lost to follow-up. At the 12-month follow-up, five participants (nine pairs) did not attend the appointments due to the COVID-19 pandemic regulations. A total of 45 children (82 pairs) were assessed and analyzed. At the 18-month follow-up, 93.81% of the pairs (91 pairs) were assessed and analyzed. Figure 1 illustrates a flowchart of the participants up to the 18-month follow-up.

### Clinical findings

Concerning the clinical examination of the 77 pairs of teeth at the 6-month follow-up, 70 pairs (90.9%) did not develop caries on either the teeth that received Vanish^TM^ XT or FV. One pair (1.3%) developed caries in both teeth. Dental caries were detected on the tooth that received FV but not on the one that received Vanish^TM^ XT in six pairs (7.8%). The difference in caries development between the Vanish^TM^ XT and FV was found to be statistically significant (*P* = 0.031) with the Vanish^TM^ XT showing better results (Table 2).

Clinical examination of the 82 pairs of teeth after 12 months evidenced that 69 pairs (84.2%) did not develop occlusal caries on either the tooth that received Vanish^TM^ XT or FV. Two pairs (2.4%) developed caries in both teeth. Ten pairs (12.2%) developed new occlusal caries in the teeth that were treated with FV only, whereas only one pair (1.2%) treated with Vanish^TM^ XT developed caries in the occlusal surface. Vanish^TM^ XT prevented occlusal caries by 90% compared to FV and the results were found to be statistically significant (*P* = 0.012) (Table 2).

**Table 2:** Clinical examination findings of pairs at the 6-, 12-, and 18-month follow-up

The caries status of the molars in the pair	6 months (*N* = 77)	12 months (*N* = 82)	18 months (*N* = 91)
Both molars in the pair were sound	70 (90.9%)	69 (84.2%)	75 (82.4%)
Both molars in the pair were carious	1 (1.3%)	2 (2.4%)	2 (2.2%)
Vanish XT molar developed caries and FV molar was sound	0	1 (1.2%)	1 (1.1%)
FV molar developed caries and Vanish XT molar was sound	6 (7.8%)	10 (12.2%)	13 (14.3%)
**Odds ratio (OR)** **(95% CI)**	NA	0.1 (0.01–0.82)	0.08 (0.01–0.63)
**P-value**	0.031*	0.012*	0.002*

* Statistically significant at *P* < 0.05, using McNemar’s test

Regarding the clinical examination at the 18-month follow-up, among the 91 pairs of teeth evaluated, 75 pairs (82.4%) did not develop occlusal caries on either the teeth treated with Vanish^TM^ XT or the FV. Two pairs (2.2%) developed caries in both teeth. Thirteen pairs (14.3%) had developed occlusal caries in the FV tooth, whereas only one pair (1.1%) in the Vanish^TM^ XT tooth developed occlusal caries. When compared to teeth treated with FV, teeth treated with Vanish^TM^ XT had 0.08 (95% CI, 0.01–0.63) the odds of developing occlusal caries (*P* = 0.002).

Radiographic examinations at 6, 12, and 18 months follow-up revealed no proximal carious lesions in either group. An intention-to-treat analysis (ITT) was performed on 53 participants (97 pairs) where pairs of participants lost to follow-up were considered failed (carious). The ITT results demonstrated that Vanish^TM^ XT continued to have superior performance compared to FV with statistical significance at 6 months (*P* = 0.018), 12 months (*P* = 0.003), and 18 months (*P* = 0.001).

## Discussion

The study was designed as a split-mouth randomized controlled clinical trial to assess the effectiveness of an RMGI cement varnish (Vanish^TM^ XT) in preventing occlusal caries in newly erupted first permanent molars, comparing its effectiveness to that of a fluoride varnish. This study was conducted over 18 months of follow-up. The sound occlusal deep pits and fissures of newly erupted permanent first molars of children at high risk of dental caries were treated with either the Vanish^TM^ XT or fluoride varnish. A split-mouth design where subjects act as their own controls was used in the current study to minimize bias, to reduce inter subject variability, to reduce random error, and to increase efficiency, accuracy and power to detect diffrences. The study focused on children between the ages of 6 and 9 because their first molars are highly susceptible to dental careis and are recommended to be sealed with fissure sealants [11, 13, 14]. However, these teeth are still in the early stages of eruption, making it challenging to isolate them. Therefore, alternative caries preventive materials such as FV or Vanish^TM^ XT are advocated. Due to the low mineralization of newly erupted teeth, it is recommended to apply sealants on these molars as soon as they erupt [15]. The morphology of the occlusal surface and position of the newly erupting molars (not in full occlusion/function) favor biofilm accumulation, which subsequently leads to caries formation, which can be prevented by sealing these sites [16].

Vanish^TM^ XT, an RMGI cement varnish, was selected as the experimental material because of innovative approach for the prevention of occlusal caries in newly erupted permanent first molars. Vanish^TM^ XT is a light-curable material with the properties of glass ionomer materials, such as remineralization and bonding. It contains fluoroaluminosilicate glass, which can release and take up fluoride for a prolonged period of time, and calcium glycerophosphate, which supplies phosphate and calcium [17]. RMGI varnishes do not require complete isolation of the tooth and can be used for newly erupted teeth. This has been used as a caries-preventive approach in various clinical studies [9, 18].

Insufficient data are available in the literature addressing the clinical effects of light-curable RMGI varnishes on the prevention of occlusal caries in newly erupted first permanent molars over different follow-up periods. To the best of our knowledge, this blinded, randomized controlled clinical trial is one of the few studies carried out to investigate the effect of Vanish^TM^ XT on occlusal caries prevention of newly erupted teeth over an 18-month follow-up period and was compared to a fluoride varnish as a control group.

In the current study, we used FV (Vanish^TM^ 5% Sodium Fluoride White Varnish) with tricalcium phosphate as a control because it is considered one of the best fluoride varnish materials in the market due to its unique formulation. This fluoride varnish is composed of tricalcium phosphate in addition to the 5% NaF, which helps in tooth remineralization. A varnish is a resin modified in an alcohol-based suspension. The FV can easily reach areas such as deep pits and fissures because of the high flowability of the material. Additionally, it can be used in both dry and wet environments. The FV had extended fluoride release compared to the other varnishes after 4 h and up to 24 h.

The study monitored the progression of caries among participants up to 18 months. In clinical practice, it is recommended that peditric patients are not supposed to forgo dental visits for a period exceeding 12 months [10], depending on their risk of developing dental caries. Therefore, we opted to conduct a follow-up for a maximum of 18 months which exceeds the recommended maximum recall visit. Although several participants did not appear for the follow-up visits, sometimes due to the COVID-19 condition, there were multiple efforts to reach them, and a few of them did appear at subsequent visits. For the purpose of evaluating and controlling for any possible bias related to loss to follow-up, an intention to treat analysis (ITT) was conducted. This analysis assumed that all participants who were lost to follow-up had developed caries on their molars, which is the worst-case scenario. Even under the most extreme conditions, the ITT findings demonstrated that Vanish^TM^ XT outperformed FV.

The current study evidenced a significantly higher caries development on the occlusal surfaces of newly erupted permanent first molars in the control group than that in the experimental group at the 6-month follow-up. This finding aligns with that of the study by Gonçalves et al. [6], in which occlusal caries development was significantly higher in the control group when compared to the Vanish^TM^ XT group after 6 months. However, in that study the control group did not receive any treatment and the type of permanent molar and eruption stage is not clear. The authors’ study sample comprised: 36 teeth treated with Vanish^TM^ XT, of which 33 were available for the 6-month follow-up; and 40 teeth without treatment, of which 28 were available for the 6-month follow-up. The study did not have a split-mouth design, and patients treated with Vanish^TM^ XT were different than those with no treatment. All these factors were considered limitations that might have affected the results [6].

In contrast, a study by Cabral et al. [9]. shows disagreement with our results. The authors found similar caries development on occlusal surfaces of partially erupted first permanent molars at the 6-, 12-, and 24-month follow-up between the Vanish^TM^ XT group and the active control group treated with high-viscosity GIC (FUJI IX), and the difference was not statistically significant. However, their study only included teeth without caries and with initial caries according to ICDAS-II criteria (codes 1, 2, and 3). Additionally, a glass ionomer sealant (FUJI IX) was used as the active control group. The clinical outcome was measured as dentin caries development on the occlusal surfaces with ICDAS- II codes 4, 5, or 6. In contrast, our study considered initial enamel caries (ICDAS-II code 1) as a failure to prevent caries. All these factors may have affected their findings [9].

The results of the current study at the 12-month follow-up revealed that there was a significantly higher number of occlusal surfaces with new carious lesions on teeth that were treated with FV compared to those that were treated with Vanish^TM^ XT. Few studies have investigated the effect of Vanish^TM^ XT on occlusal caries prevention in newly erupted first permanent molars over a 12-month follow-up period. Recent studies have contradicted our findings at 12 months of follow-up [9, 19].

In the study by Uchil et al., the effect of Vanish^TM^ XT in preventing occlusal caries in partially erupted first permanent molars was investigated. They reported no superiority of Vanish^TM^ XT in preventing occlusal caries compared to the active control upon 12 months of follow-up. However, they used a glass ionomer sealant (Fuji VII) as the active control [19].

Regarding the 18-month follow-up of the current study, FV teeth significantly developed a higher percentage of occlusal caries than the Vanish^TM^ XT group. When reviewing the literature, researches of Vanish^TM^ XT and its effect on occlusal caries prevention are limited. No study assessing the effect of Vanish^TM^ XT on occlusal caries prevention upon 18 months of follow-up was found; thus, direct comparison with our study is difficult.

Our study revealed significantly higher caries prevention with the use of Vanish^TM^ XT at 6, 12, and 18 months of follow-up compared to the fluoride varnish. This difference in caries prevention between the Vanish^TM^ XT and FV may be explained by their different mechanisms of fluoride release. Fluoride release from a NaF varnish is high following its application, and the burst effect at the initial stages lasts for various hours. Fluoride release continues until abrasion from brushing or chewing food wears away the bulk of the varnish coat, which subsequently reduces fluoride availability at the tooth surfaces [20]. In contrast, fluoride release burst with Vanish^TM^ XT lasts for various days [17]. Additionally, owing to the nature of the chemical bond and resin infiltration between the RMGI component of the Vanish^TM^ XT and the tooth structure, there is a continuous, slow, and prolonged fluoride release over the lifetime of the Vanish^TM^ XT, which is assumed to be 6 months [21]. This property of Vanish^TM^ XT plays a significant role in caries prevention [17, 22].

Based on the findings of the current study, Vanish^TM^ XT has various benefits for children, their parents, pediatric dentists, and society. Application of Vanish^TM^ XT facilitates oral hygiene, leading to less dental caries development and teeth extraction, therefore preserving oral function and aiding self-esteem, fewer visits to the pediatric dentists and lower treatment costs. For pediatric dentists, Vanish^TM^ XT is a material easy to apply that can last on teeth surfaces for 6 months, providing the preventive standard of care in fewer dental visits when compared to fluoride varnish. For the society, Vanish^TM^ XT has proven effective in caries prevention of occlusal surfaces of partially erupted molars in children at high risk of dental caries, and as it does not require a complete dental setup, it can be used to prevent dental caries in rural communities that lack access to oral health care providers.

The present study highly contributes to the literature on the effect of RMGI (Vanish^TM^ XT) in occlusal caries prevention over 6, 12, and 18 months of follow-up. This information was previously available in databases. The present study has various strengths, such as using a split-mouth design to limit the effect of confounding factors that might affect the primary outcome, using a blindness protocol to minimize bias, and implementing calibration of the dentist who perform all evaluations.

This study had some limitations. A high number of patients could not attend the 6-, 12-, and 18-month follow-up due to the COVID-19 pandemic lockdown. Moreover, owing to the nature of the treatments, it was difficult to blind the operator to the type of treatment each occlusal surface received at the time of application. Furthermore, considering that dental caries may require a significant amount of time to develop, an 18-month follow-up period may be viewed insufficient. Nevertheless, the prevalence of dental caries among children in Saudi Arabia is quite high, with rates ranging from 60 to 70% [23]. Furthermore, the period of greatest susceptibility to dental caries for the first permanent tooth occurs within the first few years following eruption. Therefore, it was determined that a period of 18 months would be sufficient for follow-up. More studies are needed to evaluate the effect of Vanish^TM^ XT in occlusal caries prevention of newly erupted first permanent molars over longer follow-up periods.

## Conclusion

Compared to fluoride varnish, the light curable RMGI Vanish^TM^ XT is significantly more effective in prevention of occlusal surfaces carious lesions in newly erupted first permanent molars at a 6-, 12-, and 18-month follow-up.

### Clinical significance

The RMGI Vanish^TM^ XT can be used as a sealant alternative material when proper isolation is challenging, such as with newly erupted molars, for occlusal caries prevention.

## Data Availability

The datasets used and/or analysed during the current study are available from the corresponding author on reasonable request.

## Abbreviations

GIC: Glass Ionomer Cement
RMGI: Resin-Modified Glass Ionomer
ICDAS-II: International Caries Detection and Assessment System
